# Influence of Alkali Activator Type on the Hydrolytic Stability and Intumescence of Inorganic Polymers Based on Waste Glass

**DOI:** 10.3390/ma15010147

**Published:** 2021-12-25

**Authors:** Adrian Ionut Nicoara, Alina Ioana Badanoiu

**Affiliations:** 1 Department of Science and Engineering of Oxide Materials and Nanomaterials, Faculty of Applied Chemistry and Materials Science, University Politehnica of Bucharest, 1-7 Gheorghe Polizu Street, 011061 Bucharest, Romania; alina.badanoiu@upb.ro; 2 Academy of Romanian Scientists, 54 Independentei, 050094 Bucharest, Romania; 3 National Research Center for Micro and Nanomaterials, University Politehnica of Bucharest, 1–7 Gheorghe Polizu Street, 011061 Bucharest, Romania

**Keywords:** fire protection, intumescent materials, inorganic polymers, alkali activator, binders, humidity

## Abstract

The main objective of this study is the synthesis and characterization of low cost alkali-activated inorganic polymers based on waste glass (G-AAIPs) using a mixture of NaOH and Ca(OH)_2_ as alkali activators, in order to improve their hydrolytic stability. This paper also presents detailed information about the influence of composition determined by X Ray Diffraction (XRD), microstructure determined by Scanning Electronic Microscopy (SEM) and processing parameters on the main properties of G-AAIP pastes. The main factors analyzed were the glass fineness and the composition of the alkaline activators. The influence on intumescent behavior was also studied by heat treating of specimens at 600 °C and 800 °C. The use of Ca(OH)_2_ in the composition of the alkaline activator determines the increase of the hydrolytic stability (evaluated by underwater evolution index) of the G-AAIP materials compared to those obtained by NaOH activation. In this case, along with sodium silicate hydrates, calcium silicates hydrates (C-S-H), with good stability in a humid environment, were also formed in the hardened pastes. The highest intumescence and an improvement of hydrolytic stability (evaluated by underwater evolution index and mass loss) was achieved for the waste glass powder activated with a solution containing 70% NaOH and 30% Ca(OH)_2_. The increase of the waste glass fineness and initial curing temperature of G-AAIPs have a positive effect on the intumescence of resulted materials but have a reduced influence on their mechanical properties and hydrolytic stability.

## 1. Introduction

Fire is a serious threat to people’s lives and property, frequently leading to the destruction of buildings with economic and architectural importance (for example, Grenfell Tower, UK 2017 or Notre Dame de Paris, FR, 2019). Therefore, protecting the infrastructure against fires is a necessity. For this purpose, different methods of protection against fire (active or passive) have been developed [1,2,3,4]. Fire can cause considerable damage to steel-based metal structures due to steel high thermal conductivity, which implies the rapid loss of resistance and rigidity at high temperatures (for example World Trade Center, SUA, 2001). Therefore, fire protection is necessary for buildings with metal structures [3,4,5].

The existing methods for passive fire protection of steel rely on the use of materials that reduce the heat transfer to the protected metal structure. Intumescent materials swell when subjected to fire and can be used as passive fire protection in buildings; these materials can provide thermal protection to the underlying structure or can seal the penetrations in walls/floors preventing fire and smoke spreading in adjacent rooms [6].

Passive fire protection materials are available in different forms: paints, panels, blocks or strips. Panels made of silicate-based intumescent materials could stop, at least for a while, the spread of fire and smoke in the event of a fire. Silicate-based materials can be used as coatings for metal structures for which overheating should be avoided. Potential applications of these types of intumescent materials include lining ventilation ducts and service wells in tall buildings, and so forth [7].

Intumescent silicate-based materials can be produced by the alkaline activation with NaOH solutions of waste glass powder with/without different admixtures [8,9,10]. As a result, municipal waste glass can be upcycled in the production of this type of materials. Although glass can be used several times without significant changes in its chemical and physical properties, glass recycling, except for the transparent one, remains negligible [11]. According to EU data, European countries recycle over eight million tons of glass containers annually. However, waste glass must fulfill a series of requirements if is intended to be recycled in the manufacture of other glassware. The trend is to collect and sort urban and industrial glass waste by type (there are already special containers for collecting neon tubes and special containers for colored glass bottles). Even so, the mixing of different types of glass with different chemical compositions and different particle size distributions involves recycling through complex technological processes. As a result up to 30% of the glass waste can no longer be recycled and it is necessary to look for alternative ways of valorization [11,12]. The use of glass waste as supplementary cementitious material in the cement production or as aggregate in concrete production was extensively studied [12,13,14,15,16,17,18]. Scientific works [19,20,21,22,23] have also demonstrated the feasibility of using municipal and industrial waste glass as a source of silica for the complete or partial replacement of sodium silicate in the alkaline activation of slag and/or fly ash.

Alkali-activated inorganic polymers (AAIP) with good mechanical properties but with poor hydrolytic stability were obtained using as solid precursors waste glass (cullet) [24,25,26]. This limitation is due to the reduced hydrolytic stability of the sodium silicate hydrates that are formed by the interaction of the glass powder with the alkaline activator [10,25,26].

Menchaca-Ballinas and Escalante-Garcia [27,28] reported the improvement of underwater stability of materials obtained by the alkaline activation (with CaO and NaOH) of waste glass and limestone filler mixtures. This promotes the formation of calcium silicate hydrates which are more stable in the wet environment than sodium silicate hydrates. 

Thus, the main challenge in obtaining intumescent AAIPs, based on waste glass powder by an alkali activation process, is to achieve an optimal correlation between composition-structure-properties; these materials should conserve their intumescent properties when cured in various environments and should have adequate mechanical strengths, in correlation with their utilization.

The main objective of this study is the synthesis and characterization of low cost alkali-activated inorganic polymers based on glass waste (G-AAIPs) using a mixture of NaOH and Ca(OH)_2_ as alkali activators, in order to improve their hydrolytic stability evaluated by underwater evolution index (u.e.i.) This paper presents also information about the influence of composition determined by X ray diffraction (XRD), microstructure determined by Scanning Electronic Microscopy (SEM) and processing parameters on the main properties of AAIP pastes. Compression strength, hydrolytic stability and intumescent properties at temperatures of 600–800 °C of all samples were also evaluated.

## 2. Materials and Methods

**Figure 1:**
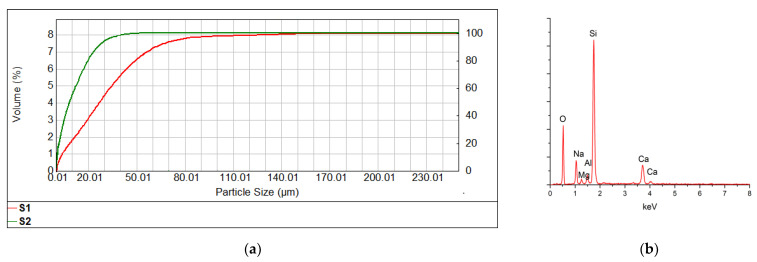
Particle size distribution (**a**) of S1 glass powder (red line) and S2 glass powder (green line) and EDS spectrum of glass powder (**b**).

In order to obtain glass alkali-activated inorganic polymers (G-AAIP) pastes, glass cullet from glass bottles (various colors) were used as raw material. The glass powder was obtained by grinding glass culets in a planetary ball mill. Two types of glass powders were obtained: S1 glass powder with a Blaine specific surface area of 2086 cm^2^/g and glass powder S2 with Blaine specific surface area of 2998 cm^2^/g. The particle distributions of these glass powders are presented in Figure 1a and information regarding the elemental composition of glass, assessed by EDS, are present in Figure 1b.

As can be seen in Figure 1a, S2 glass powder contains a higher number of particles of smaller sizes (d_90_ = 25.87 µm) as compared with the coarser ground glass (S1). S1 glass powder has a much wider particle size distribution, also containing particles with sizes larger than 100 μm. For S1 glass powder d_90_ is 62 µm.

As expected, the EDS spectrum of soda-lime-glass showed the presence of Si, O, Ca and Na (Figure 1b). 

The G-AAIP samples were obtained by mixing the glass powder with an alkaline activator solution (Table 1); the solid and liquid components were mixed with a mechanical stirrer (at 800 rpm for 10 min) and the obtained paste was poured into cylindrical molds with 34 mm diameter and 15 mm height. The samples were cured in two different conditions:

- in the air at 25 °C, R.H. = 50%,
- in air at 60 °C for first 24 hours and then in the air at 25 °C, R.H. = 50%.

The mineralogical composition of G-AAIP pastes was assessed by X-ray diffraction analysis (XRD); this analysis was performed at room temperature using a Panalytical Empyrean diffractometer with Cu Κα (λ = 0.154 nm) radiation, and the scanning was performed between 2θ = 10–80°.

The microstructure of the studied materials was assessed using a Quanta Inspect F50 high-resolution electronic scanning microscope (1.2 nm resolution-Thermo Fisher—former FEI, Eindhoven, The Netherlands) with an energy-dispersive spectrometer (SEM-EDS).

The compressive strengths of G-AAIP pastes, hardened for different periods of time and in different conditions, was assessed with a testing machine (Shimatzu, Japan); the loading was performed at a rate of 0.1 mm/min, on triplicate specimens cured in similar conditions.

In order to evaluate the intumescent proprieties of studied G-AAIPs, the paste specimens, previously hardened for 28 days, were thermally treated at 600 °C and 800 °C for 60 minutes, in an electrical furnace using a heating rate of 10 °C/min.

The volume and mass changes of specimens determined by the thermal treatment were calculated using Formulas (1) and (2) [29].

ΔV = [(Vf − Vi)/Vi] × 100 [%],(1)

where: Vi = specimen’s volume before heat treatment; Vf = specimen’s volume after heat treatment.

Δm = [(mf − mi)/mi] × 100 [%],(2)

where: mi = specimen’s mass before thermal treatment; mf = specimen’s mass after thermal treatment.

In order to evaluate the hydrolytic stability of the G-AAIP pastes, three specimens, previously cured for 28 days, were immersed and kept in distilled water for 90 days; water volume to specimen volume ratio was three. The other three specimens were kept the same period of time in the air at room temperature (25 ± 2 °C).

The electrical conductivity of the solution in which the specimens were immersed for 90 days was assessed with a laboratory multiparameter InoLab Multi 9630 IDS (WTW, Weilheim, Germany).

The mass change of specimens cured for 90 days immersed in water was calculated with the formula:

ΔM = [(Mw − Md)/Md] × 100 [%],(3)

where: Md = specimen’s mass after 28 days of curing in air; Mw = specimen’s mass after immersion in water for 90 days and drying.

To quantify the influence of wet environment on the mechanical properties of the studied G-AAIPs, the underwater evolution index (u.e.i) was calculated using the formula proposed by Menchaca-Ballinas and Escalante-Garcia [28].

u.e.i. = under water compressive strength/dry compressive strength.(4)

## 3. Results

**Figure 2:**
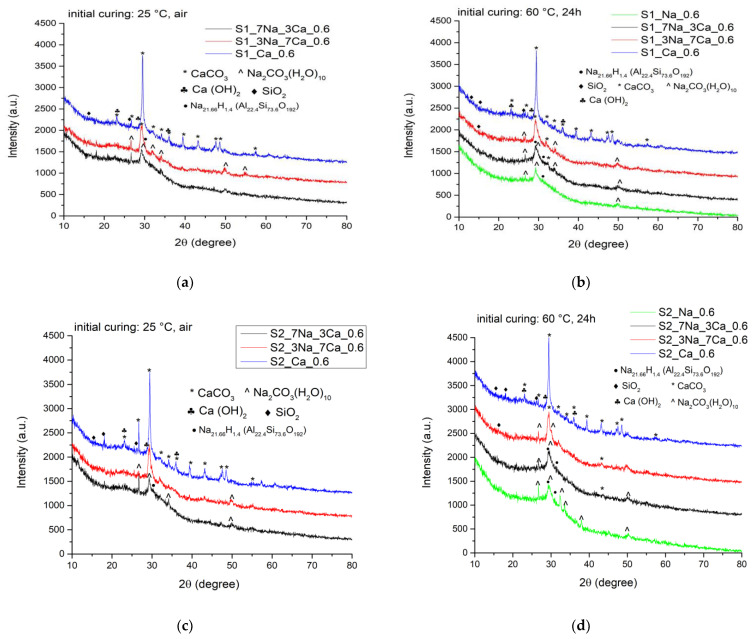
XRD patterns recorded after 7 days of hardening for: (**a**) samples obtained with S1 glass powder cured at 25 °C; (**b**) samples obtained with S1 glass powder cured first at 60 °C for 24 h and then at 25 °C; (**c**) samples obtained with S2 glass powder cured at 25 °C; (**d**) samples obtained with S2 glass powder cured first at 60 °C for 24 h and then at 25 °C.

The mineralogic compositions of G-AAIP samples after 7 days of hardening were assessed by X-ray diffraction (XRD). The XRD patterns presented in Figure 2 show a diffraction halo between 15–35 degrees which indicates the presence, in all samples, of compounds with a low crystallinity degree (gels) formed as a result of the interaction between glass and alkaline activators-Ca(OH)_2_ or/and NaOH. The main reaction products formed by the alkaline activation of glass powder with NaOH solution are sodium silicate (aluminate) hydrates (N-S-(A)-H) [24,26,27,28]; if the alkaline activator is Ca(OH)_2_ or mixtures of NaOH and Ca(OH)_2_, the main hydrates formed are calcium silicate hydrates (C-S-H) and calcium aluminate silicate hydrates (C-A-S-H) [28]. After 7 days of hardening, the crystallinity degree of these hydrates is low therefore their assessment on XRD patterns is difficult. The peaks present on the XRD patterns of specimens cured for 7 days (Figure 2) suggest the presence of CaCO_3_ (identified by the PDF 04-008-0198) resulted by the carbonation of nonreacted Ca(OH)_2_.

It should be noted that the samples activated only with sodium hydroxide and cured for 7 days at 25 °C were not hard enough to be analyzed by XRD. The curing at 60 °C in the first 24 h determined the increase of the hardening rate of this type of materials; therefore, the XRD analysis of these specimens was possible. The XRD patterns of S1_Na_0.6 and S1_7Na_3Ca_0.6 suggest the presence of N-S-H gel along with a small amount of sodium carbonate (probably formed by the carbonation of non-reacted NaOH).

The increase of the glass powder fineness (pastes prepared with S2—Figure 2c,d) does not seem to have a noticeable influence on the amount and crystallinity degree of the reaction products.

**Figure 3:**
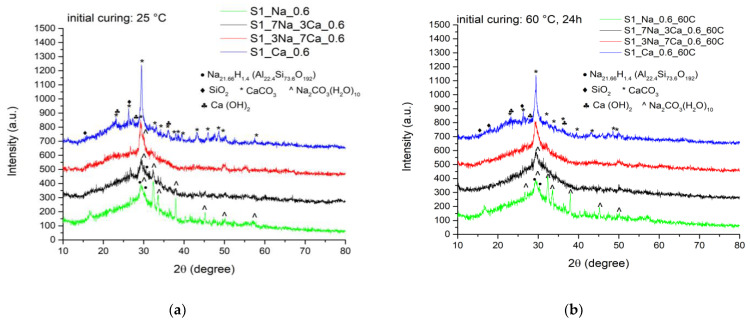
XRD patterns recorded after 28 days of hardening for samples obtained with S1 glass powder stored at 25 °C (**a**) and cured first at 60 °C for 24 h and then at 25 °C (**b**).

After 28 days of hardening, one can notice a certain increase of crystallinity degree of compounds present in the hydrated pastes, demonstrated by the higher intensities of the diffraction peaks. The curing in air of samples increases the carbonation phenomenon, especially for samples with a higher content of sodium hydroxide (Figure 3).

**Figure 4:**
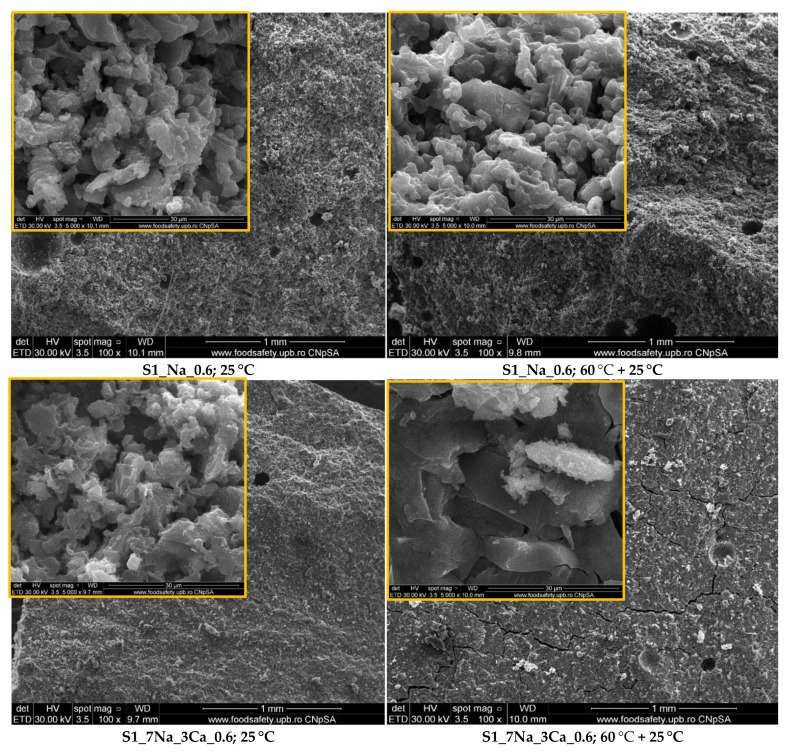

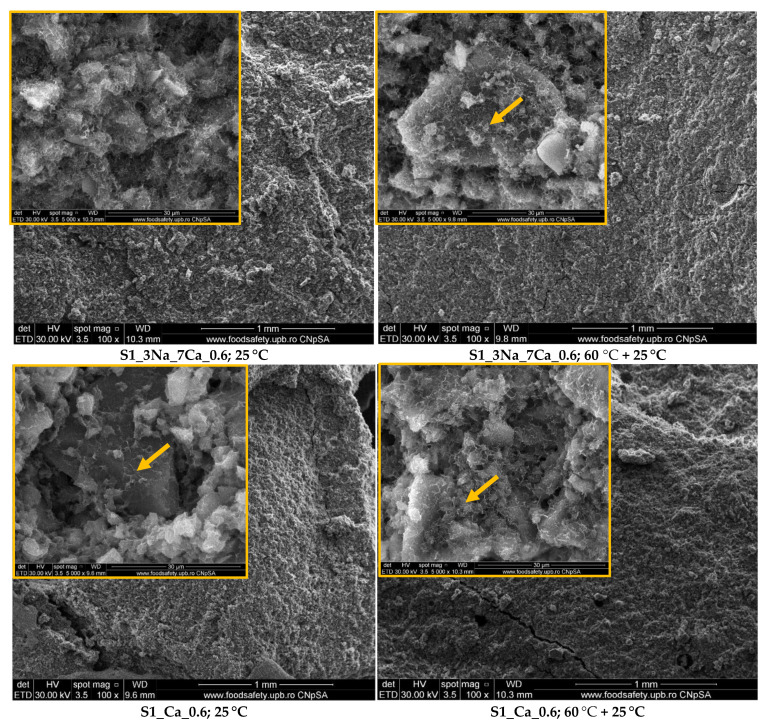
Electronic microscopy analyses (SEM) of samples based on S1 glass powder stored at 25 °C or at 60 °C for the first 24 h and then at 25 °C.

**Figure 5:**
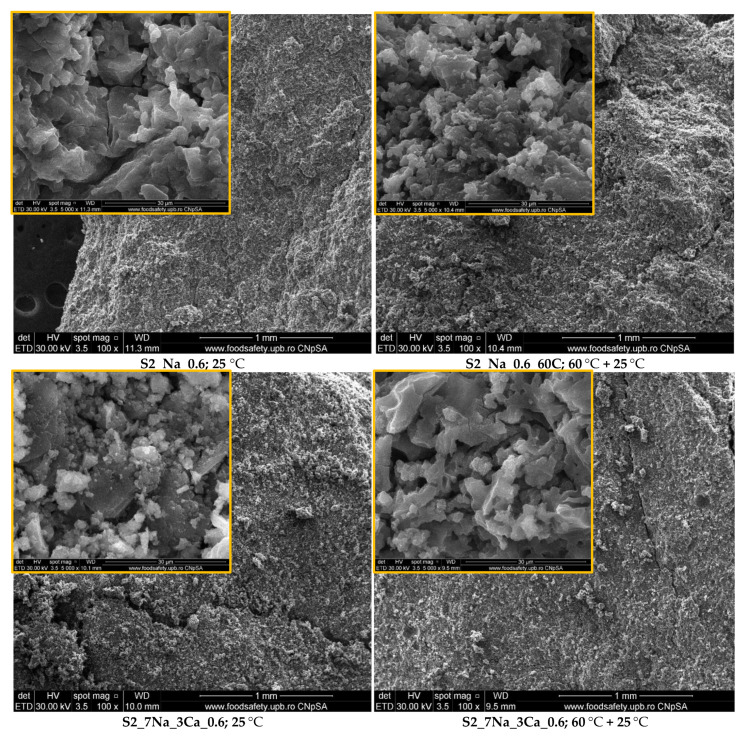

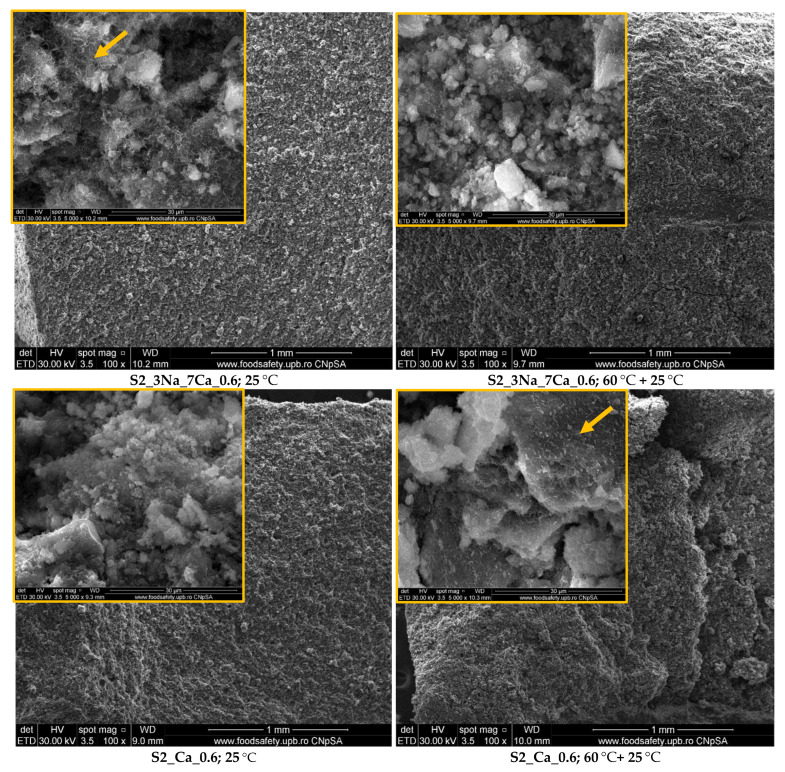
Electronic microscopy analyses (SEM) of samples based on S2 glass powder stored at 25 °C or at 60 °C for the first 24 h and then at 25 °C.

Figure 4 and Figure 5 show the microstructures of G-AAIPs hardened in different conditions. On the SEM images one can assess two types of morphology: well-definite rectangular shapes characteristic for sodium silicate hydrates (see inserts on SEM images for samples S1_Na_0.6—Figure 4 and S2_Na_0.6—Figure 5) and fibrillar and foil-like structures specific to C-S-H (see arrows) in rich calcium samples (S1_Ca_0.6 and S1_3Na_7Ca_0.6—Figure 4).

According to Abdollahnejad et al. [30], the calcium silicate hydrate gel coexists with the geopolymeric gel (resulted from the alkaline activation of an aluminosilicate source), and the incorporation of Ca^2+^ into the geopolymeric network acts as a charge balancing action. 

The initial curing at 60 °C for 24 h does not change the microstructure of the compounds formed by alkaline activation but improved the compressive strengths assessed over a shorter period of hardening (7 days), especially for the G-AAIPs prepared with a higher amount of NaOH.

**Figure 6:**
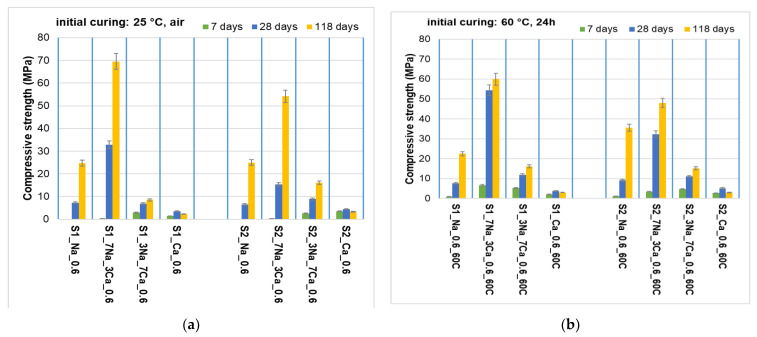
Compressive strengths vs. time for: (**a**) samples obtained with S1 and S2 glass powders stored at 25 °C; (**b**) samples obtained with S1 and S2 glass powder cured at 60 °C for the first 24 h and then stored at 25 °C.

The compressive strengths of the obtained G-AAIPs, after different periods of times and in different curing regimes, are presented in Figure 6.

The use of the 70%NaOH + 30%Ca(OH)_2_ mixture as an alkaline activator led to the highest compressive strengths values. The lower values obtained for the G-AAIPs based on S2 glass power, as compared with those based on S1, can be explained by the decrease of the fresh paste workability; this is due to the increase of the fineness of the glass powder and to the use of the same liquid to solid ratio as in the case of specimens based on coarser S1 waste glass powder. Even if the reactivity of the glass powder is higher (due to its higher fineness) and more hydrates are formed by alkaline activation, this positive effect is outweighed by the negative effect of a worse workability and compaction [27].

**Figure 7:**
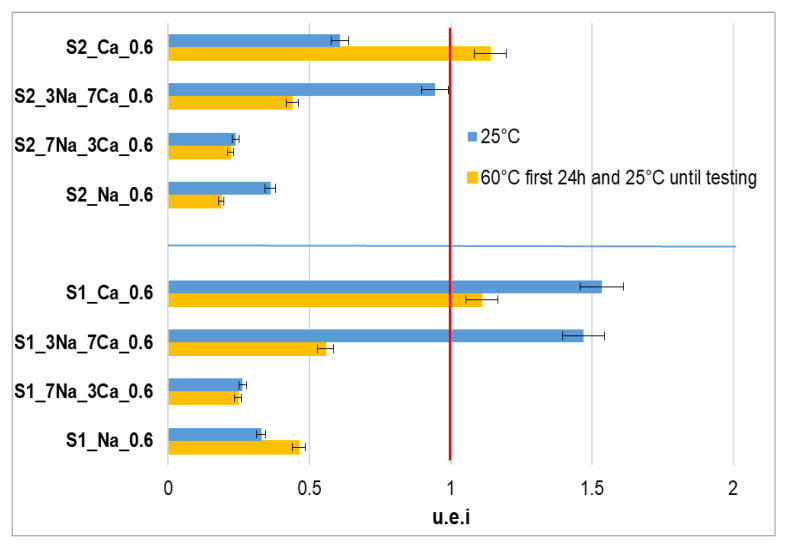
Underwater evolution index (u.e.i) for samples obtained with S1 respectively S2 glass powders stored the first 28 days at 25 °C (blue) or at 60 °C for the first 24h and then at 25 °C (orange) followed by curing underwater or air curing for the next 90 days.

Underwater evolution index (u.e.i) is a direct measure of the effect exerted by the sample’s storage in water on the mechanical properties. If u.e.i has values higher than 1, the compressive strengths of the samples cured under water are higher as compared with those of the samples cured in air. As can be seen from Figure 7, for some samples activated with Ca(OH)_2_ solution, the value of this coefficient is higher than 1 and is explained by the presence of a high amount of calcium silicate hydrates (with good stability in humid medium). On the contrary, the specimens activated with NaOH solution have small values of u.e.i. due to the low hydrolytic stability of sodium silicate hydrates [26,27].

**Figure 8:**
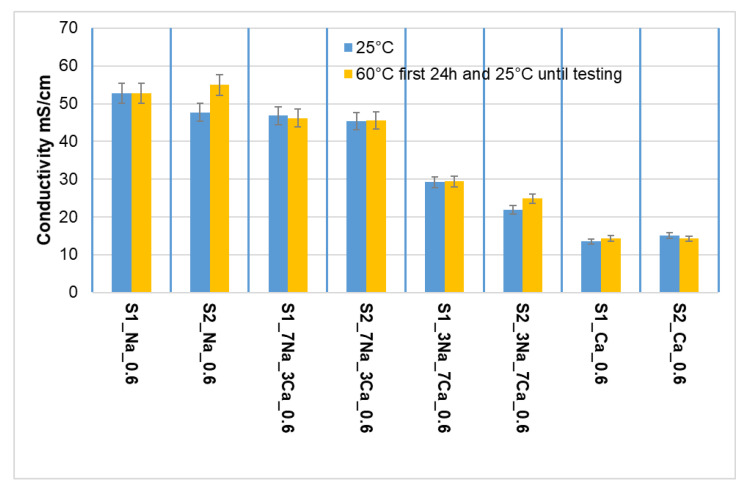
The electrical conductivity of the solutions resulting from the storage for 90 days in distilled water of G-AAIPs prepared with glass powders with different fineness (S1—2086 cm^2^/g and S2—2998 cm^2^/g).

**Figure 9:**
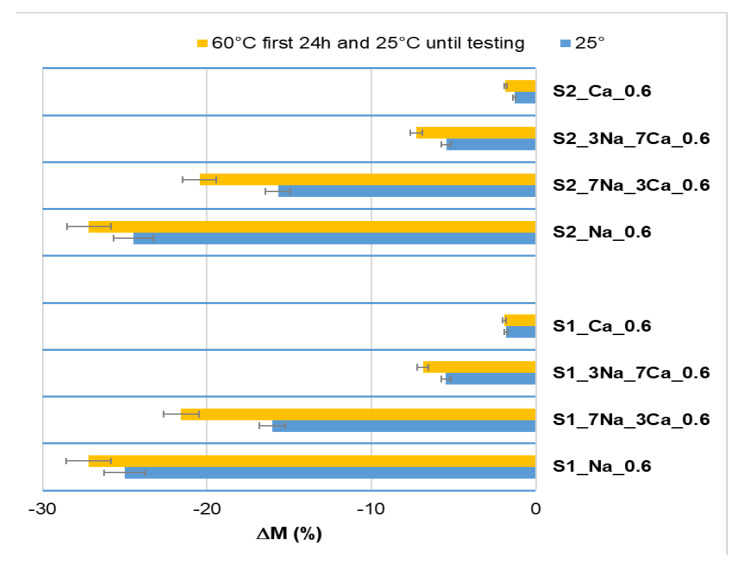
The mass loss of G-AAIPs obtained from glass powders with different fineness (S1—2086 cm^2^/g and S2—2998 cm^2^/g) after immersion in distilled water for 90 days.

As can be seen in Figure 8, the increase of NaOH amount in samples’ composition leads to an increase of the electrical conductivity of aqueous solution resulting when the specimens were cured underwater for 90 days; this is mainly explained by the high solubility of sodium silicate hydrates. On the contrary, the solutions resulting when the specimens activated with Ca(OH)_2_ were cured underwater have lower values of electrical conductivity in correlation with the lower solubility of C-S-H [26,27]; the higher hydrolytic stability of the specimens prepared with Ca(OH)_2_, is confirmed by G-AAIPS mass losses (Figure 9). The higher values of mass loss recorded for the specimens activated with NaOH solution confirm their low hydrolytic stability and the improvement of this property when Ca(OH)_2_ or mixtures of Ca(OH)_2_ and NaOH are used for the activation of glass powder.

The mass losses recorded for the specimens activated with NaOH solution and cured the first day at 60 °C are higher as compared with those of the specimens cured at 25 °C. This can be explained by the increase of the reaction rate and formation of a higher amount of sodium silicate hydrates when the initial curing temperature is 60 °C.

The materials obtained by the alkaline activation of waste glass powder with NaOH solution exhibit intumescent behavior [8,9,10]. In order to quantify the influence of the use of Ca(OH)_2_ as an alkaline activator on the intumescence of these materials, samples were thermally treated in air at 600 °C and 800 °C for 1 h.

**Figure 10:**
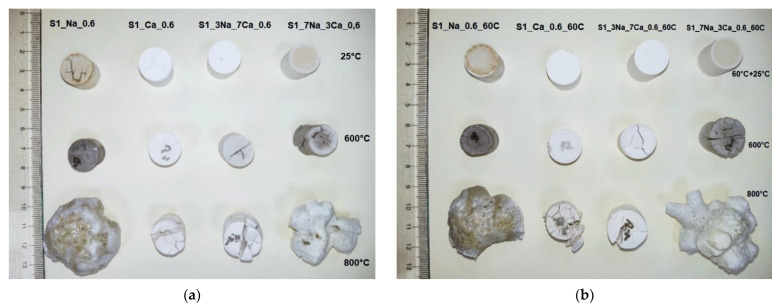

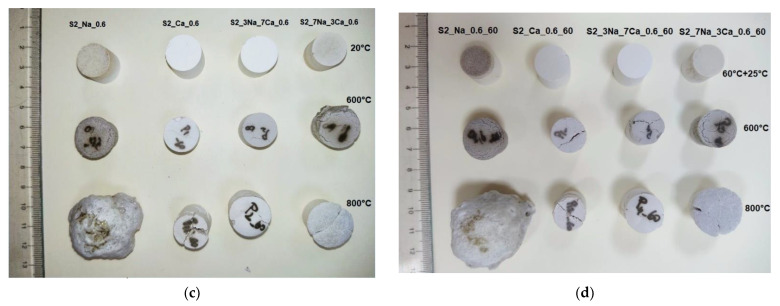
The visual aspect of GAAIPs before and after thermal treatment at 600 °C and 800 °C: (**a**) samples obtained with S1 glass powder stored at 25 °C, (**b**) samples obtained with S1 glass powder cured at 60 °C for 24 h and then stored at 25 °C (**c**) samples obtained with S2 glass powder stored at 25 °C (**d**) samples obtained with S2 glass powder cured at 60 °C for 24 h and then stored at 25 °C.

**Figure 11:**
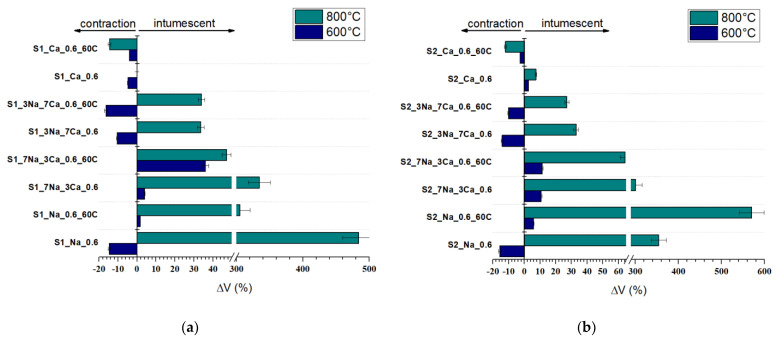
Volume change vs. temperature for (**a**) G-AAIPS based on S1 glass powder and (**b**) G-AAIPS based on S2 glass powder.

Figure 10 presents the visual aspect of G-AAIPs before and after thermal treatment, and Figure 11 presents the specimens’ volume changes.

As can be noticed (Figure 10), the thermal treatment at 600 °C determined the shrinkage of G-AAIPs obtained using Ca(OH)_2_ as an alkaline activator. For the specimens obtained with NaOH and mixtures of NaOH and Ca(OH)_2_, one can notice a volume increase due to the intumescent phenomenon, when the thermal treatment is performed at 800 °C.

The increase of NaOH content in G-AAIPs determines an increase of volume (ΔV- Figure 11). This behavior is determined by the higher amount of sodium silicate hydrates formed in these specimens; the water loss and transformation of N-S-H during the thermal treatment contribute to the volume (and porosity) increase [8].

The use of Ca(OH)_2_ as alkaline activator leads to C-S-H formation. C-S-H dehydration during the thermal treatment determines the formation of cracks (visible also on the specimens’ surface—Figure 10), mainly due to the pressure caused by water evaporation [31].

**Figure 12:**
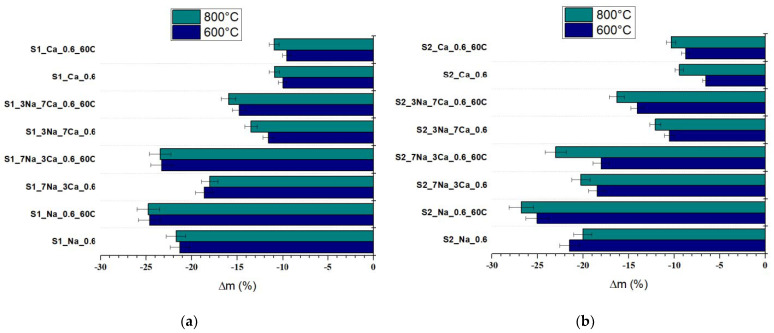
Mass changes vs. temperature for (**a**) samples obtained with S1 glass powder and (**b**) samples obtained whit S2 glass powder.

All G-AAIPs also exhibit mass losses when thermally treated at 600 °C and 800 °C (Figure 12); the increase of NaOH amount and initial curing temperature determines the increase of mass losses in correlation with the nature and amount of formed hydrates.

## 4. Conclusions

Intumescent materials can be obtained by the alkali activation of waste glass power with NaOH. Due to the nature of formed hydrates (mainly sodium silicate hydrates), the hydrolytic stability of these materials is poor. One possible way to improve this property is to use Ca(OH)_2_ as an alkaline activator. In this case, the resulting calcium silicates hydrates (C-S-H) have a good stability in a humid environment but at the same time exert a negative influence on the intumescence of this type of material. 

The highest intumescence and an improvement of hydrolytic stability (underwater stability) was achieved for the waste glass powder activated with a solution containing 70% NaOH and 30% Ca(OH)_2_. 

The increase of the waste glass fineness and of the initial curing temperature had a positive effect on the intumescence phenomenon of the resulting G-AAIPs but have a marginal influence on their mechanical properties and hydrolytic stability. 

One potential utilization for these materials is as intumescent panels or blocks designed to seal, in the event of a fire, the penetration in walls and floors, thus preventing the spread of fire and smoke in the building.

## Figures and Tables

**Table 1:** Compositions of studied G-AAIP pastes.

Sample	Glass Powder (%wt.)		NaOH (%wt.)	Ca(OH)_2_ (%wt.)	Water (%wt.)	NaOH + Ca(OH)_2_ to Glass Powder Ratio	Liquid to Solid Ratio
	S1	S2					
S1_Na_0.6	57.1	-	8.6	-	34.3	0.15	0.60
S2_Na_0.6	-	57.1	8.6	-	34.3	0.15	0.60
S1_7Na_3Ca_0.6	57.1	-	6.0	2.6	34.3	0.15	0.60
S2_7Na_3Ca_0.6	-	57.1	6.0	2.6	34.3	0.15	0.60
S1_3Na_7Ca_0.6	57.1	-	2.6	6.0	34.3	0.15	0.60
S2_3Na_7Ca_0.6	-	57.1	2.6	6.0	34.3	0.15	0.60
S1_Ca_0.6	57.1	-	-	8.6	34.3	0.15	0.60
S2_Ca_0.6	-	57.1	-	8.6	34.3	0.15	0.60

## Data Availability

Data sharing not applicable.
